# MicroRNA-182 targets protein phosphatase 1 regulatory inhibitor subunit 1C in glioblastoma

**DOI:** 10.18632/oncotarget.21309

**Published:** 2017-09-27

**Authors:** Liqiang Liu, Xiaowei Zhang, Chengrui Nan, Zongmao Zhao, Shucheng Ma, Wenhua Li, Hongchao Hu, Zhaohui Liang

**Affiliations:** ^1^ Neurosurgical Department, The Second Hospital of Hebei Medical University, Shijiazhuang, Hebei, China; ^2^ Neurosurgical Department, Dongying People’s Hospital of Shandong Province, Dongying, Shandong, China

**Keywords:** MicroRNA-182, glioblastoma, PPP1R1C, protein phosphatase 1 regulatory inhibitor subunit 1C

## Abstract

Glioblastoma (GBM) is an incurable cancer, with mean post-diagnosis survival time of 14-16 months. Metagenomic analysis by The Cancer Genome Atlas (TCGA) program has identified microRNA-182-5p (miR-182-5p or miR-182) as the only miRNA associated with favorable disease prognosis and temozolomide (TMZ) susceptibility. Previous reports have indicated that miR-182 down regulates expression of *BCL2L12, c-MET*, and *HIF2A*. However, other messenger RNA (mRNA) targets of miR-182 have not been validated which would explain its association with a favorable disease prognosis. *In situ* analysis revealed that protein phosphatase 1 regulatory inhibitor subunit 1C (*PPP1R1C*) is a putative target of miR-182. PPP1R1C protein and RNA expression as assessed by tissue microarray and quantitative real time PCR, respectively, was inversely correlated to miR-182 expression in glioblastoma patients and in the metastatic glioblastoma cell line U87-MG. Reporter assays using *PPP1R1C* 3′ untranslated region (UTR) showed that miR-182 can interact with the wild-type but not a miR-182-5-seed mutant. Ectopic expression of miR-182 mimic in the U87-MG cell line significantly decreased proliferation as well as suppressed *in vitro* migration and invasion. Opposite observations were made when the non-malignant neuronal cell line HCN-2 was transfected with anti-miR-182 antagomir. The miR-182 mimic or siRNA targeting *PPP1R1C* induced TMZ susceptibility indicating that decreased susceptibility to TMZ in GBM patients might be attributed to high expression of PPP1R1C. Inverse correlation of *PPP1R1C* mRNA and miR-182 levels in 20 GBM patients confirmed the same. Cumulatively, our results indicate that loss of miR-182 leads to increased expression of PPP1R1C which in part explain disease progression and resistance to TMZ therapy.

## INTRODUCTION

MicroRNAs (miRNAs) are a group of endogenous, small noncoding RNA. MiRNAs have been well characterized and are known to functionally regulate diverse cellular and pathogenic processes. MiRNAs can either bind or degrade their target mRNA or inhibit their translation; the decision to degrade mRNA or inhibit translation being determined by the degree of complementarity to the target transcript’s sequence [1]. More importantly, miRNAs have been shown to function both as tumor suppressors or oncogenes [2], even though the precise context dependent ques that make them function like this are currently unknown. Given their important role in tumorigenesis *per se*, a lot of research has focused on the role of miRNAs in glioblastoma (GBM).

GBM is one of the most debilitating cancers with mean survival time around 14-16 months post-diagnosis [3]. Of 470 miRNAs profiled by The Cancer Genome Atlas (TCGA) program, only miR-182 expression level has been shown to be associated with better prognosis, suggesting miR-182 might function as a tumor suppressor in the case of GBM [4]. It has been shown that miR-182 targets *BCL2L12, c-MET and HIF2A* in GBM and thus integrates growth, apoptosis, and differentiation programs in GBM [5]. However, our understanding of the expression and function of miRNA-182 in GBM pathogenesis is still limited. It is thus imperative to develop a more complete picture about how suppression of miR-182 observed in GBM potentiates the disease.

Our *in situ* analysis and subsequent experiments revealed that protein phosphatase 1 regulatory inhibitor subunit 1C (*PPP1R1C*) is a target of miR-182 in GBM and up regulation of *PPP1R1C* in GBM patients is partially responsible for observed resistance to temozolomide (TMZ) susceptibility.

## RESULTS

### Differential expression of miR-182 and PPP1R1C in glioblastoma cell lines

**Figure 1:**
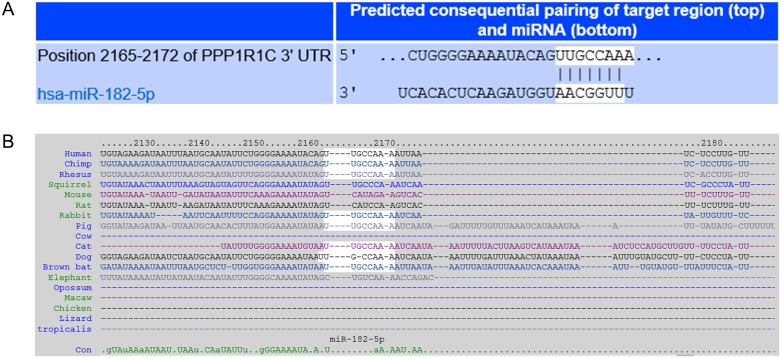
Prediction of *PPP1R1C* as a target of miR-182 **(A)** Complementary 7mer-m8 seed match between miR-182 and the 3′ UTR of *PPP1R1C* as predicted by TargetScan software. **(B)** Conservation of the 7mer-m8 seed of miR-182 in the 3′UTR of *PPP1R1C* in indicated organisms.

To explore the mechanism of miR-182-mediated regulation in GBM, we used two independent algorithms to *in situ* predict the targets for miR-182, TargetScan and microCosm. Among the predicted genes, were the previously validated *Bcl2L12*, *c-Met* and *HIF2A* [5]. In addition, one of the putative targets identified by both software was *PPP1R1C* (encoding the protein phosphatase 1 regulatory inhibitor subunit 1C) or protein phosphatase-1 (PP1) (Figure 1A and *data not shown*). The miR-182 seed region in the 3′UTR of *PPP1R1C* was conserved across different species (Figure 1B). PP-1 has been previously indicated to promote tumor growth in cervical cancer [6, 7]. However, no previous knowledge is available of PP-1 being targeted by miRNAs, hence we decided to pursue it further.

**Figure 2:**
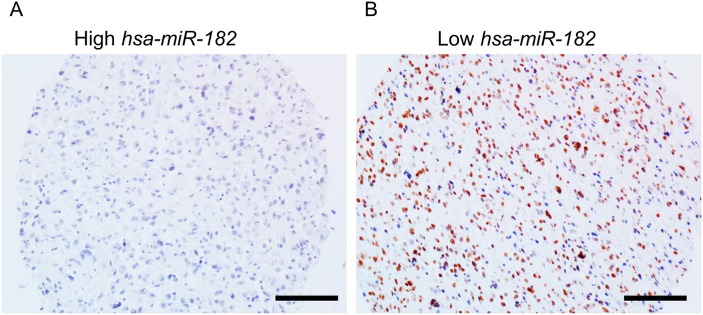
*PPP1R1C* and miR-182 are inversely correlated in patients with GBM Representative immunohistochemistry images showing *PPP1R1C* expression in glioblastoma tissue with differential expression levels of miR-182 expression as determined by qRT-PCR. Brown staining represents positive *PPP1R1C* expression. Scale bar – 40 μm.

We next determined miR-182 levels in tumor tissue specimens obtained from 20 GBM patients by qRT-PCR. Representative sections with low (17/20) and high (3/20) relative miR-182 expression, compared to matched non-malignant tissue, were evaluated for PPP1R1C expression by immunohistochemistry. As shown in Figure 2, tissue specimens with high miR-182 expression were negative for PPP1R1C staining (Figure 2A), whereas those with low miR-182 expression had robust PPP1R1C staining (Figure 2B). Of note, since the number of patients with high miR-182 expression were too few for inclusion in analysis to calculate statistical significance of the observed inverse correlation between miR-182 and PPP1R1C expression.

### PPP1R1C is a bona-fide target of miR-182

**Figure 3:**
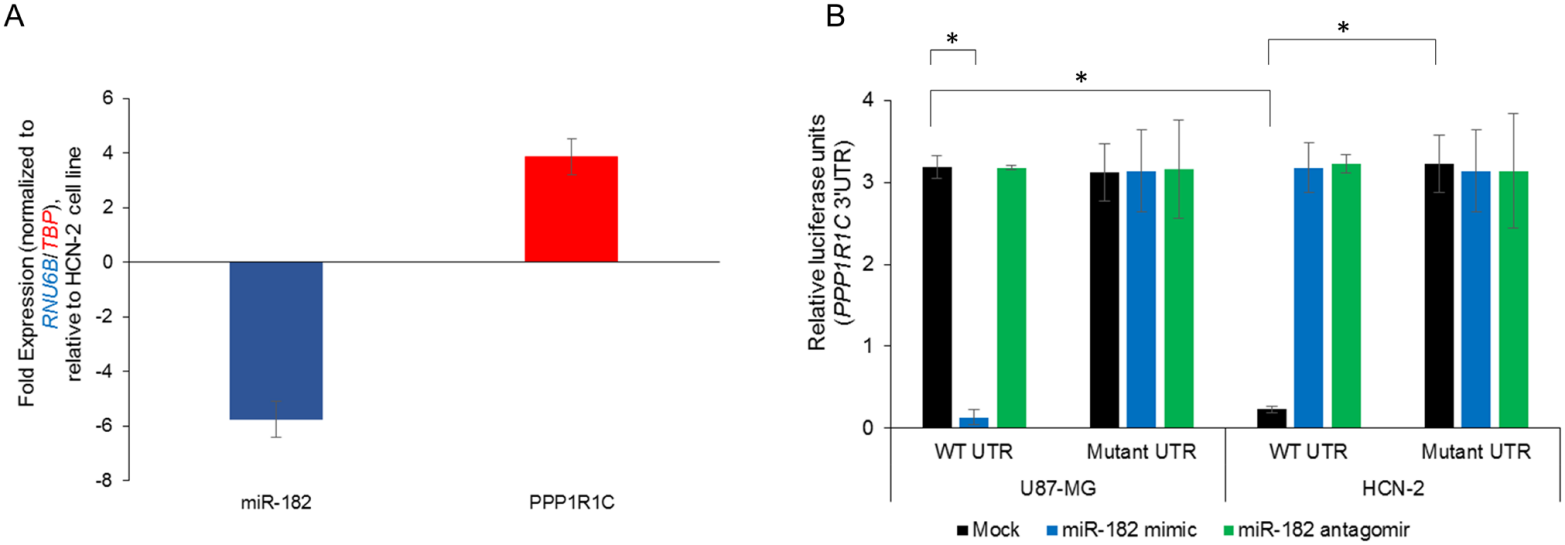
*PPP1R1C* is a bona-fide target of miR-182 in the glioblastoma cell line U87-MG **(A)** Steady state expression of miR-182 and *PPP1R1C* in U87-MG and HCN-2 cell lines were determined. Data was normalized to *RNU6B* and *TBP* expression, respectively. Fold expression in U87-MG cells was determined relative to expression in HCN-2 cell line. **(B)** Relative luciferase activity of transiently transfected luciferase reporter constructs containing either full-length or mutated (miR-182 binding site deleted) *PPP1R1C* 3′ UTR in U87-MG and HCN-2 cells, either mock transfected or transfected with miR-182 mimic or miR-182 antagomir. ^*^p<0.05. Data in ‘A’ and ‘B’ represent at least three independent experiments, each done in triplicate.

We next determined if *PPP1R1C* is a bona fide target of miR-182. We initially evaluated relative miR-182 and *PPP1R1C* expressions in the glioblastoma cell line U87-MG and the non-malignant HCN-2 cell line. U87-MG cell line had a significantly suppressed miR-182 expression (−5.76 ± 0.42 folds) and significantly increased *PPP1R1C* expression (+3.86 ± 0.67 folds) compared to the HCN-2 cell line (p<0.05 in each case) (Figure 3A). To test putative interaction between the 3′UTR of *PPP1R1C* and miR-182, luciferase reporter constructs containing the wild-type *PPP1R1C* 3′UTR were transfected in HCN-2 and U87-MG cells alone or in combination with miR-182 mimic or miR-182 antagomir (Figure 3B). Expression of the wild-type *PPP1R1C* 3′UTR containing reporter were inhibited 2.89 ± 0.03 folds (P = 0.0003) in HCN-2 cells compared with the U87-MG cells. Expression of the wild-type *PPP1R1C* 3′UTR containing reporter were inhibited 3.2 ± 0.05 folds (P = 0.004) in U87-MG cells transfected with the miR-182 mimic in compared to the no-mimic control. Expression of the wild-type *PPP1R1C* 3′UTR containing reporter was restored in HCN-2 cells transfected with miR-182 antagomir (3.43 ± 0.35 folds (P = 0.007) compared to the no-antagomir control. To confirm that the effects observed was due to miR-182 targeting the *PPP1R1C* 3′UTR, we generated and tested a miR-182 binding mutant of the *PPP1R1C* 3′UTR reporter (nucleotides 2165-2172 corresponding to miR-182 binding site). Whereas, the miR-182 binding mutant *PPP1R1C* reporter did not show any difference in relative luciferase activity between mock and miR-182 mimic transfected in U87-MG cells (p>0.05), the repression observed with wild-type reporter was completely ablated following mutation in the HCN-2 cell line (Figure 3B), cumulatively confirming that *PPP1R1C* mRNA is a *bona-fide* target of miR-182 in these cells.

### Effect of differential PPP1R1C expression on malignant behavior of glioblastoma cells

**Figure 4:**
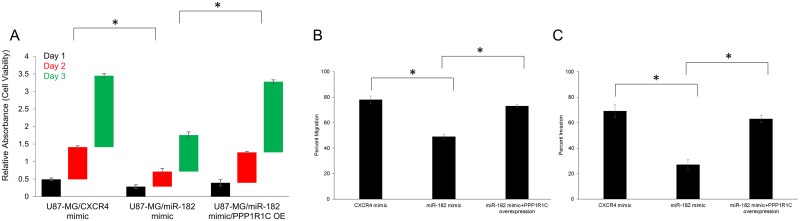
*PPP1R1C* expression levels dictate cell viability, migration and invasion abilities in GBM cells **(A)** Cell viability was measured in U87-MG cells transfected with CXCR4 mimic, miR-182 mimic, or miR-182 mimic along with *PPP1R1C* overexpression (OE) at 24, 48, and 72 hours after transfection by the MTT assay. **(B, C)** Modulation of miR-182 changes cell migration and invasion abilities in the U87-MG cells. The migrated and invasive cells were photographed using a microscope, and the number of the migrated and invasive cells in every field was counted and represented as percent of total cells at the beginning of the assay. Error bars, S.D. ^*^p<0.05. In each panel, data represent at least three independent experiments, each done in triplicate.

To determine whether *PPP1R1C* expression affects cell proliferation in GBM, cell viability assays were carried out with U87-MG cells, transfected with either CXCR4 mimic or miR-182 mimic. Transfection with miR-182 mimic suppressed cell proliferation after day 1 (CXCR vs miR-182 mimic – 0.49 ± 0.04 vs 0.29 ± 0.05, p<0.05), day 2 (CXCR vs miR-182 mimic – 0.92 ± 0.04 vs 0.43 ± 0.09, p<0.05), and day 3 (CXCR vs miR-182 mimic – 2.04 ± 0.06 vs 1.04 ± 0.09, p<0.05) (Figure 4A). MiR-182 have other targets like *BCL2L12* and *HIF2A*, modifications of which can result in the same observation. Hence, to confirm that the observed effects on cell proliferation were due to *PPP1R1C*, we rescued *PPP1R1C* expression by overexpressing the coding sequence of *PPP1R1C* in U87-MG cells transfected with the miR-182 mimic. Overexpression of *PPP1R1C* significantly rescued the suppression of cell proliferation (day 1: 0.39 ± 0.04; day 2: 0.87 ± 0.04; day 3: 2.01 ± 0.06) (p<0.05) (Figure 4A). Our results thus confirmed that *PPP1R1C* potentiates cell proliferation in GBM.

We scored each of the individual transfectants U87-MG cells (CXCR4 mimic, miR-182 mimic, and miR-182 mimic with *PPP1R1C* overexpression (OE)), for migration (Figure 4B) and invasion (Figure 4C) in standard transwell assays. Using these criteria, phase contrast imaging and quantification showed that overexpression of miR-182 inhibited *in vitro* migration (CXCR vs miR-182 mimic – 78% ± 3% vs 49% ± 2%, p<0.05), and invasion (CXCR vs miR-182 mimic – 69% ± 5% vs 27% ± 4%, p<0.05). Overexpression of *PPP1R1C* significantly rescued the migration (73% ± 4%) and invasive (63% ± 6%) potential of U87-MG cells transfected with the miR-182 mimic (p<0.05). Our results suggested that *PPP1R1C* potentiates *in vitro* migration and invasion in GBM, which occurs by repression of miR-182 expression. Cumulatively, the profound repression in relative expression of miR-182 and increased expression of *PPP1R1C* in GBM tissue or cell line along with its capacity to impinge *in vitro* migration and invasion suggested that it may drive tumorigenesis and metastatic progression in GBM [8].

### MiR-182 replenishment or knock-down of PPP1R1C increases chemosensitivity of glioblastoma cells

**Figure 5:**
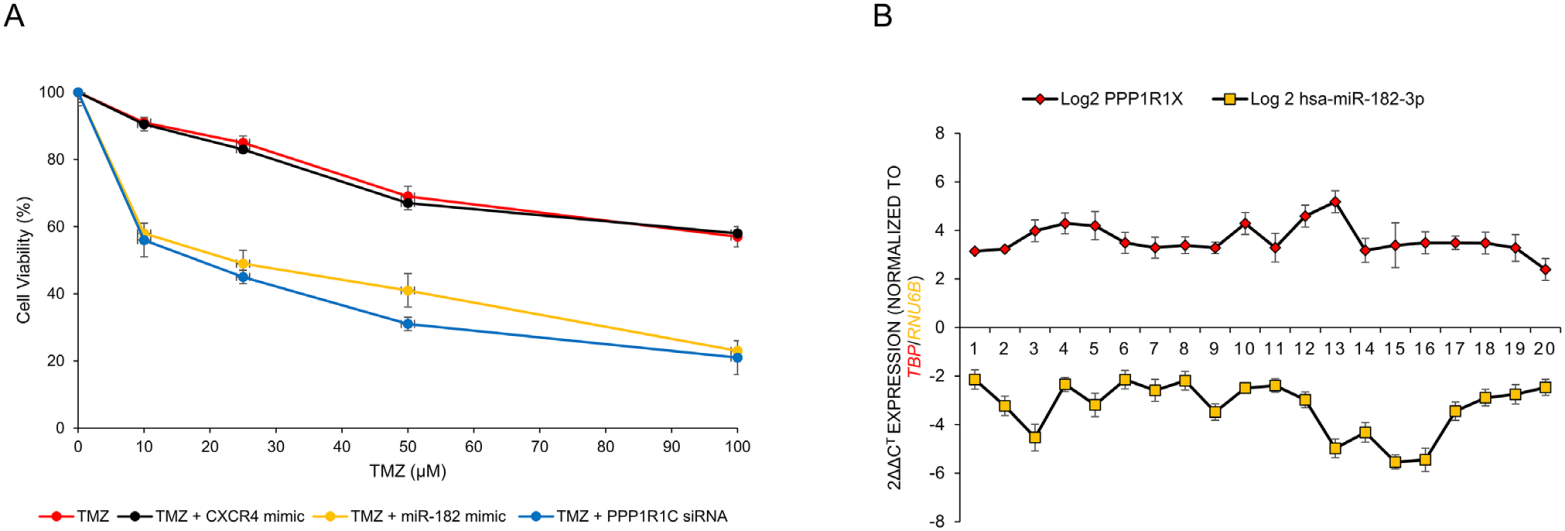
MiR-182 and PPP1R1C levels are inversely correlated in glioblastoma patients and their modulation can be used to increase sensitivity to temozolomide (TMZ) **(A)** U87-MG cells were either untransfected or transiently transfected with either CXCR4 mimic, miR-182 mimic, or siRNA against *PPP1R1C* for 12 hours. The cells were then treated with indicated doses of TMZ for 72 hours. Cell viability was assessed by the MTT assay. Each experiment was carried out at least 3 times. Data represent mean ± SEM. Data represent at least three independent experiments, each done in triplicate. **(B)** MiR-182 and *PPP1R1C* mRNA are inversely correlated in patients with glioblastoma. Pearson correlation demonstrating the inverse relation between miR-182 and *PPP1R1C* in paired samples (P < 0.05, Pearson correlation r = −0.8761). Data represent at least three independent experiments, each done in triplicate.

To determine the therapeutic potential of miR-182 and *PPP1R1C* as a chemosensitizer, we evaluated the effect of miR-182 mimic-mediated replenishment and *PPP1R1C* siRNA-mediated depletion on the cytotoxicity of TMZ on U87-MG cells. Transfection of miR-182 mimic significantly chemosensitized U87-MG cells (IC_50_ from 69 ± 3 μM in TMZ alone to 41 ± 1 μM in TMZ+miR-182 mimic, p<0.05). The *PPP1R1C* siRNA chemosensitized the cells even more (IC_50_ from 69 ± 3 μM in TMZ alone to 31 ± 3 μM in TMZ+*PPP1R1C* siRNA, p<0.05) (Figure 5A). Cumulatively, these results indicate that miR-182 replenishment or *PPP1R1C* depletion sensitizes GBM cells to the cytotoxicity of TMZ.

Given that our experiments indicated that *PPP1R1C* is a bona-fide target of miR-182, we hypothesized that suppression of miR-182 expression might be an underlying feature of GBM pathogenesis. We determined miR-182 and *PPP1R1C* expression in 20 GBM patients. Our results indicated a dynamic and inverse correlation between down-regulation in the levels of miR-182 and the observed increase in the *PPP1R1C* in GBM tissue specimens (Figure 5B) (P <.005, Pearson correlation r = −0.8761).

## DISCUSSION

In the current study, our experimental results show that miRNA-182 and *PPP1R1C* is downregulated and upregulated, respectively, in human GBM tissues. MiRNAs are evolutionarily conserved 21-23 nucleotides RNAs that regulate post-transcriptional gene expression either by blocking translation or degrading target messenger RNAs (mRNAs) and have been increasingly shown to function as tumor suppressors or oncogenes [9, 10]. MiRNAs can function in both normal and transformed cells and have even been shown to play a role in metastasis [11–14].

It has been recently shown that the gene repressors, HDAC1 and HDAC2, became recruited to the promoter of miR-182 and represses it in acute myeloid leukemia [15]. In addition, it was shown that HDAC inhibitors could de-repress miR-182 expression which increased sensitivity of AML cells to sapacitabine treatment [15–16]. Given that miR-182 has now been shown to target *Bcl2L12, c-Met and HIF2A* in GBM [5] and *PPP1R1C*, also in GBM, in this study, it would be interesting to evaluate HDAC inhibitors in GBM treatment. An alternative will be a combination treatment with TMZ and HDAC inhibitors to potentiate optimal treatment outcome.

Repression of *PPP1R1C* by using miRNA-182 mimic inhibited cell proliferation, as well as migration and invasion. Finally, modulating miR-182 or *PPP1R1C* levels increased sensitivity of GBM to TMZ treatment. Cumulatively, this highlights miR-182 and *PPP1R1C* as potential biomarkers in GBM. It will be clinically relevant and interesting to determine if exosome-mediated delivery of miR-182 will be able to restore miR-182 anti-oncogenic function in GBM cells, and whether such a design can potentially substitute or better currently utilized TMZ therapy in GBM patients.

It will be interesting to investigate in future research endeavors how miR-182 and *PPP1R1C* expression varies and correlates to disease progression in GBM patients that had either surgical resection, radiation therapy, or adjuvant chemotherapy. Not a lot is known about function of PPP1R1C in normal brain tissue or in cancer. There has been one finding of a *CREB-PPP1R1C* gene fusion associated with enrichment of insulin signaling pathway genes in breast cancer [17]. RNA-seq analysis in GBM cells overexpressing PPP1R1C will provide important indicators about function of PPP1R1C in GBM pathogenesis.

It will also be of potential interest to study if miR-182 mediated control of *PPP1R1C* is also functionally important in cervical cancer pathogenesis [6, 7]. Conversely, it will be important to verify if other known targets of miR-182, like *FOXO1* in prostate cancer, are also deregulated in GBM. A complete understanding of miR-182 mediated regulation might emerge from experiments looking at gene expression following overexpression and knockdown of miR-182 in GBM cells.

## MATERIALS AND METHODS

### Tissue samples, processing, and ethical considerations

Fresh-frozen and paraffin-embedded GBM tissue specimens and corresponding adjacent non-malignant brain tissue samples were obtained from 20 Chinese patients at the Second Hospital of Hebei Medical University between 2014 and 2015. All cases were included post review by pathologist and only where complete clinical pathology and follow-up data was available. None of the 20 included patients underwent pre-operative local or systemic treatment. The study protocol was approved by the Institutional Review Board of the Second Hospital of Hebei Medical University. Freshly harvested samples were immersed in RNAlater (Life Technologies, Shanghai, China) before snap freezing within 30 minutes post-surgery. All tissue samples were stored in liquid nitrogen until further use.

### Cell culture

The non-malignant encephalitis cell line HCN-2 and the glioblastoma cell line U87-MG line were obtained from ATCC (Beijing, China) and cultured in DMEM medium, containing 10% FBS (Lonza, Germany), and 100 U/mL penicillin and 0.1 mg/mL streptomycin. Cells were maintained at 37°C under a humidified atmosphere of 5% carbon dioxide.

### RNA and miRNA extraction and quantitative real time polymerase chain reaction (qRT-PCR)

Total RNA was isolated from cultured cells and tumor tissues using Trizol reagent. First strand cDNA was synthesized using the RevertAid™ First Strand cDNA synthesis Kit (Life Technologies, Shanghai, China), which was then used for real-time polymerase chain reaction (PCR) using TaqMan Gene Expression probes (Life Technologies, Shanghai, China). *TBP* (TaqMan Assay ID: Hs00427620_m1) was used as an internal control for assessing *PPP1R1C* (TaqMan Assay ID: Hs00976529_m1) transcript level. Data was normalized to *TBP* expression and analyzed by the -ΔΔCt method. According to the manufacturer’s instructions, miRNA from tissues and cells was extracted using the mirVana miRNA isolation kit (Life Technologies, Shanghai, China), and the expression levels of hsa-miR-182 and *RNU6B* were detected by TaqMan miRNA assays (Life technologies, Shanghai, China, TaqMan Assay IDs: 002334 and 001093, respectively). Data was normalized to *RNU6B* expression and analyzed by the -ΔΔCt method.

### Gene construction

The *PPP1R1C* 3′ UTR clone in pMirTarget was obtained from Origene. The *PPP1R1C* 3′ UTR reporter was constructed by amplifying the endogenous *PPP1R1C* 3′ UTR from the Origene. XhoI and ApaI sites were added to the 5′ and 3′ ends of the fragment during the preceding PCR reaction and cloned into the XhoI and ApaI site on the Rr-luc-6XCXCR4 Renilla luciferase vector (Addgene). To make the *PPP1R1C* 3′UTR mutant construct, site-directed mutagenesis was used to delete 2165-2172 region, corresponding to the hsa-miR-182 binding site. A firefly luciferase vector was used as transfection and normalization control in all luciferase assays. The *PPP1R1C* plasmid encoding the coding sequence was obtained from BioClone Inc. (USA). Constructs were sequence verified before being used in experiments.

### Transfection and luciferase assays

HCN-2 and U87-MG cells (4 × 10^4^) were transiently transfected using Lipofectamine LTX (Life Technologies, Shanghai, China) as per the manufacturer’s instructions. Where indicated, cells were transfected with CXCR4 or miR-182 mimic or antagomir (Life Technologies, Shanghai, China) along with the *PPP1R1C* 3′UTR constructs. In indicated cases, the plasmid encoding the coding sequence of *PPP1R1C* was co-transfected with the miR-182 mimic in the U87-MG cells. Ninety-six hours after transfection, the renilla and firefly luciferase activities were measured consecutively using Dual-luciferase reporter assay system (Promega, Madison, Wisconsin, USA) as per manufacturer’s protocol. Each reporter plasmid was transfected at least twice in triplicate. Post-normalization the data was represented as relative fluorescence units (RFU) ± standard deviation (SD).

### Cell proliferation assay

Cell proliferation in indicated cells was quantitated using a mitochondrial colorimetric assay (MTT assay, Sigma-Aldrich, St. Louis, MO, USA) as per the manufacturer’s recommendations. Results were expressed in terms of relative optical density (OD), as mean ± standard deviation.

### *In vitro* transwell migration and invasion assays

Migration and invasion analysis in indicated cells was done using Culturex 96 well cell migration and Culturex 96 well BME cell invasion assay kits following the manufacturer’s recommendations (R&D Systems, Shanghai, China), respectively. Data obtained from both sets of experiments were used to analyze percent migration and invasion and were expressed as mean ± standard deviation.

### Immunohistochemistry

This part of the study was approved by the Institutional Review Board of the Second Hospital of Hebei Medical University. Brain tissue specimens obtained from 20 patients with glioblastoma were first evaluated for miR-182 expression by qRT-PCR as described above. Tissue specimens representative of low and high miR-182 expression were stained for PPP1R1C expression (Abcam, Waltham, MA, USA; Catalogue ab111224). The stained slides were scored by a pathologist as positive and negative staining blinded to the identity of the tissue cores.

### Drug treatment

U87-MG cells were either not transfected or transfected with either of CXCR4 mimic, miR-182 mimic, or siRNA targeting *PPP1R1C* (Silencer Select, Life Technologies, Shanghai, China) as described above. Twelve hours after transfection, the cells were subjected to treatment with indicated concentrations of temozolomide (TMZ) (Sigma Aldrich, Shanghai, China) for 72 hours. Following treatment cell viability was measured by the MTT assay as described above.

### Statistical analyses

Statistical analyses were performed using SPSS version 20.0 (IBM Corporation, NY, USA). Two-sided P-values < 0.05 were considered statistically significant.

## Abbreviations

GBM: Glioblastoma
TCGA: The Cancer Genome Atlas
TMZ: temozolomide
*PPP1R1C*: protein phosphatase 1 regulatory inhibitor subunit 1C
UTR: untranslated region
PP1: protein phosphatase-1
PCR: polymerase chain reaction
RFU: relative fluorescence units
SD: standard deviation
OD: optical density.
